# Psychometric Properties of the Interpersonal Styles Questionnaire for Physical Education in a Mexican Sample

**DOI:** 10.3390/ijerph17186636

**Published:** 2020-09-11

**Authors:** Héctor Duarte-Félix, Jorge Zamarripa, Raúl Baños, Manuel de la Cruz-Ortega, Maritza Delgado-Herrada

**Affiliations:** 1 Unidad Académica Benito Juárez, Universidad Estatal de Sonora, Fraternidad, Centro, Villa Juárez, Sonora C.P. 85294, Mexico; hector.duarte@ues.mx; 2 Facultad de Organización Deportiva, Universidad Autónoma de Nuevo León, Cd. Universitaria, s/n, San Nicolás de los Garza, Nuevo León C.P. 66451, Mexico; 3 Facultad de Ciencias de la Actividad Física y Deporte—INEF, Universidad Politécnica de Madrid, C.P. 28040 Madrid, Spain; raulfb89@gmail.com; 4 Unidad Académica Hermosillo, Universidad Estatal de Sonora, Estadio Héroe de Nacozari, Hermosillo, Sonora C.P. 83170, Mexico; manueldelacruz@ues.mx; 5 Facultad de Psicología, Universidad Autónoma de Nuevo León, Ave. Mutualismo 207-A, Mitras Centro, Monterrey C.P. 64460, Mexico; maritza.delgadohe@uanl.edu.mx

**Keywords:** self-determination theory, autonomy support, controlling style, physical education, invariance, gender, Mexico

## Abstract

During physical education classes, one of the contextual factors that can influence motivation is the teacher’s interpersonal style. The aim of this study was to analyze the psychometric properties, structure, and factorial invariance across gender of the physical education teachers’ Interpersonal Styles Questionnaire of Sonora, Mexico. The participants were 500 students (50.8% boys, 49.2% girls) aged between 9 and 13 years old (mean age (*M*_age_) = 10.72; standard deviation (SD) = 0.74) from different elementary schools of Sonora, Mexico. In terms of measuring the teacher’s interpersonal styles, the short version of the Learning Climate Questionnaire was used to measure autonomy support, whereas the Teacher Controllingness Scale was used to measure controlling style. The results support the structure and factorial invariance across gender groups of the Mexican version of the Interpersonal Styles Questionnaire for Physical Education (Cuestionario de Estilos Interpersonales en la Educación Física (CEI-EF, by its initials in Spanish)). In conclusion, the CEI-EF is a valid and reliable instrument that can be used to assess the teachers’ interpersonal styles and draw comparisons between groups of boys and girls.

## 1. Introduction

In Mexico, as in many other countries, promoting active and healthy lifestyles in children and adolescents is a stated goal of physical education programs in elementary education [1,2]. The high rates of physical inactivity and obesity seen in the population [3], together with the number of children and adolescents that could be reached, make Physical Education (PE) the appropriate means to promote health and fitness habits from an early age [4,5].

Accordingly, the behavior and motivational style of the PE teacher is an important aspect to consider, provided that the layout and the inception of positive exercise experiences fall to the teacher and have a significant impact on children with regard to adopting a physically active style during adulthood [6].

## 2. Self-Determination Theory

One theoretical framework that has examined the impact of social context on people’s behavior in different contexts, including PE [7], is the Self-Determination Theory (SDT) [8,9,10]. The core aspect of the SDT lies in the degree to which people fulfill their basic psychological needs for autonomy (the individual need to experience self-realization and choice-making opportunities), competence (an individual’s need to feel that they possess the appropriate capacity to carry out their actions), and relatedness (desire to feel involved or have a sense of belonging around others), and in the influence from the social context over their fulfillment and/or frustration. Satisfying those needs comes with an increase in confidence and a healthy motivational orientation that leads to health improvements and facilitates the development of enjoyment, determination, perseverance, commitment, and well-being [11].

Scientific literature has revealed a close relationship between the fulfillment of basic psychological needs and self-determined motivation, and the enjoyment and effort when carrying out activities [10]. In this regard, recent studies have concluded that self-determined motivation and the fulfillment of basic psychological needs (autonomy, competence, and satisfaction) in adolescents foretell physical activity habits in their leisure time at a moderate and vigorous intensity [12,13]. Therefore, students with higher levels of fulfillment of their basic psychological needs are more likely to acquire higher levels of moderate and vigorous physical activity than adolescents feeling frustrated [14]. However, the high levels of physical inactivity among adolescents during their leisure time are concerning [15], and even more among Mexican students [3] and their lack of intention to engage in physical activity in the future [16].

The interpersonal style of authority figures [17] is one of the social factors that play an important role within the educational environment, not only in performance, but also in motivation and psychological experiences resulting from student participation. One of the factors that can influence the student’s motivation in the PE classroom is the teacher’s interpersonal style, ranging from pursuing extreme control to fostering support for the adolescent’s autonomy [18,19].

An autonomy-supportive style exists when the teacher embraces behaviors deemed an important source for the fulfillment of needs while positively impacting the quality of the students’ motivation. Such behaviors include encouraging intrinsic motivation, providing a rationale for assignments, using informative/non-controlling language, showing patience with the student’s learning process, and identifying and acknowledging expressions of negative affectivity or misbehavior from the students [20].

On the contrary, a controlling style refers to the act of pursuing control over others’ behaviors [21]. This style involves behaving in a coercive manner, exerting pressure, and authoritatively imposing a specific way of behaving and thinking; it relies on intimidation and a negative conditional form of attention, rewards the execution of certain tasks, uses controlling language, and commands the lives of others, inhibiting the fulfillment of needs and thus becoming a potential source of frustration [8,22].

Several studies on the relationship between interpersonal styles and their consequences have indicated that a prevailing autonomy-supportive style comes with a series of positive effects for the teacher, such as a better functioning personality, and a more positive, affectionate, and receptive attitude toward others [23,24,25,26]. On the contrary, when a controlling style prevails, the teacher’s personality becomes negatively related to hostility, interpersonal aggression, and mistrust in social relations [27,28]. In this sense, students with higher levels of self-regulation have been traced to teachers with a more autonomous-supportive style [29].

In this way, the interpersonal style of the PE teacher influences the motor and affective experiences perceived by the students, caused both by the teacher–student interaction and by the contents presented in the classroom [30]. Thus, the teaching strategies used by teachers and the types of interaction can influence the competence of adolescents to carry out activities and achieve results, leading in turn to a change in behavior, either positive or negative, with respect to the proposed objective [31].

The interactions between teachers and students can increase positive experiences in the classroom and satisfaction with PE [32], both variables being of great concern in the physical education classrooms of Mexico [16,23].

To measure the students’ perceptions on the motivational style of their teachers, Reeve and Halusic [20] unified the short version of Learning Climate Questionnaire (LCQ-S) of Williams and Deci [33] with the Teacher Controllingness Scale (TCS) of Jang et al. [34]. The result was a 10-item instrument, six of which measure the autonomy-supportive style and the remaining four controlling style.

Other authors, such as Cheon et al. [35] used the LCQ-S [33] to assess perceptions of autonomy-supportive teaching. The authors modified the LCQ-S slightly, replacing “My teacher” with “My physical education teacher” (e.g., “My physical education teacher provides me with choices and options”). The LCQ-S values were internally consistent throughout each student’s assessment (Time 1, α = 0.83; Time 2, α = 0.88; Time 3, α = 0.93). To assess perceptions on controlling style teaching, students filled the four-item TCS [34] with slight modifications, i.e., replacing the phrase “My teacher” with “My physical education teacher” (e.g., “My physical education teacher puts a lot of pressure on me.”). The TCS scores yielded acceptable levels of internal consistency (T1, α = 0.74; T2, α = 0.80; T3, α = 0.84). It has been proven that the TCS scores are negatively correlated with those from the LCQ-S [34].

On the other hand, Behzadnia et al. [36] analyzed the scale of interpersonal styles (LCQ-S and TCS). The confirmatory factor analysis (CFA) tested the two-factor model proposed for the teachers’ interpersonal styles. Initially, the fit indexes were unsatisfactory, χ^2^_(24)_ = 93.89; *p* < 0.001; root mean square error of approximation (RMSEA) = 0.15; RMSEA 90% confidence interval (CI) = 0.12–0.18; comparative fit index (CFI) = 0.83; standardized root mean square residual (SRMR) = 0.14. This issue referred to the loading of one the TCS items on the latent construct (“My physical education instructor tries to control everything I do”). After discarding this element from the analysis, the indexes yielded a good fit, χ^2^_(18)_ = 28.02; *p* = 0.29; RMSEA = 0.06; RMSEA 90% CI = 0.00–0.11; CFI = 0.97; SRMR = 0.07. All item loadings in the final model were above 0.36, *p* < 0.01.

The study carried out by Fin et al. [18] used the modified version for physical education [37] of TCS [34]. This scale comprises of four items preceded by the phrase “My physical education teacher…” to assess the teacher’s control during the class (e.g., “attempts/tries to control everything I do”). The answers were given using a Likert scale, with scores ranging from 1 (completely disagree) to 7 (completely agree). Cronbach’s alpha was 0.86. All standardized loadings ranged between 0.27 and 0.57. In terms of the CFA, the value of χ^2^ and the fit indexes were: χ^2^_(199, 2)_ = 0.13 (*p* = 0.94), RMSEA = 0.00 (0.00, 0.04) and CFI = 0.99.

To date, no studies in Mexico are known to have analyzed the psychometric properties of both scales together, and due to the relation that different styles have with the basic psychological needs and the more self-determined motivational regulations according to the vision of SDT [8,9,10], the aim of this study was to analyze the psychometric properties, structure and factorial invariance across gender of the PE teachers’ Interpersonal Styles Questionnaire (Cuestionario de Estilos Interpersonales en la Educación Física (CEI-EF, by its initials in Spanish)) of Sonora, Mexico.

## 3. Materials and Methods

### 3.1. Study Design

This was a quantitative study with an instrumental design to assess the psychometric properties of the scale that measures the perception of autonomy-supportive and controlling interpersonal styles of the teacher in the context of physical education [38].

### 3.2. Participants

Considering the characteristics of the study and the availability of access to schools, the participants were selected using non-probability convenience sampling, considering public schools and the different grades of elementary schools of Sonora, Mexico. The final sample was 500 students (50.8% boys, 49.2% girls, mean age (*M*_age_) = 10.72; standard deviation (SD) = 0.74; range = 9–13 years old). The majority were sixth grade students (52%) from morning-session schedules (66.4%) at federal elementary schools (92.6%), with mostly male teachers (82.8%) and a high number of students having expressed being involved in sport activities (86.4%).

### 3.3. Instruments

The short version of the Learning Climate Questionnaire (LCQ-S) [33] was used to measure the student autonomy-supportive interpersonal style; this questionnaire is based on the Health-Care Climate Questionnaire [39] and has been translated into Mexican Spanish and adapted for the physical education class. The questionnaire is comprised of six items that measure the students’ perception of the support for autonomy as shown by the teacher. The instrument opens with the following header: “During my physical education class…” (“*En mi clase de educación física…*”). An example of an item is “…my teacher tries to understand how I see things before suggesting a new way to do things” (“*...mi profesor trata de comprender cómo veo las cosas antes de sugerir una nueva forma de hacerlas*”). Answers were given on a seven-point Likert scale (1 = “completely disagree”; 7 = “completely agree”).

On the other hand, the Teacher Controllingness Scale [34] was used to measure the controlling style. The scale is comprised of four items preceded by the header: “During my physical education class…” (“*En mi clase de educación física…*”). An example of an item is “…my teacher tries to control everything I do” (“*...mi profesor trata de controlar todo lo que hago*”). Answers were given on a seven-point Likert scale (1 = “completely disagree”; 7 = “completely agree”).

### 3.4. Procedure

This study was carried out according to the ethical guidelines recommended by the American Psychological Association (APA). Authorization was requested in writing to the school zone authorities and to each of the school principals, outlining the research purposes and the procedure to follow along with a model of the instrument. Afterwards, an authorization for application was requested from group teachers and the students selected according to the main inclusion criteria: to be full time students in their respective grades, to attend a regularly scheduled PE class at least twice a week, to voluntarily agree to fill the questionnaire, and to present a document duly signed by the parents or guardians acknowledging to have received the necessary information and giving their consent to participate in the research. Students were informed of the study’s goal, its voluntary nature, the absolute confidentiality of the answers and handling of data. They were also advised that there were no right or wrong answers and were asked to be completely sincere and honest. The questionnaire’s implementation was anonymous and collectively self-administered in a classroom during school hours. To homogenize data collection conditions, surveyors received background training beforehand. The protocol was approved by the Ethics Committee of the Faculty of Sports Organization of Autonomous University of Nuevo Leon (No. REPRIN-FOD-70). All subjects gave written informed consent in accordance with the Declaration of Helsinki.

The instruments were translated into Mexican Spanish using the back-translation method [40]. The translation was conducted by a professional translation agency hired by the team in charge of the study. For its reworking into the physical education framework, a group of experts (comprising of two PhD with previous experience validating psychological instruments, a physical education teacher, and a translator specializing in the area of physical education and sports) discussed the discrepancies in the translation until a first version of the instrument in Mexican Spanish was agreed upon. This version was translated back into English by a professional translation agency different to the one previously hired, and then both versions of the instrument were compared: the original source and the translation. The inconsistencies of each version were analyzed again, and certain changes were made to facilitate the comprehension of the items, attaining a final version for each of the scales. This version was presented on a pilot basis to a group of 72 students from different grades to verify that each item was comprehensible; as per the results of the pilot implementation, no comprehension issues were found. The items that comprise the scale are presented in Table 1.

### 3.5. Data Analysis

Descriptive analyses were performed for the whole scale and its constituent factors. The instrument’s structure was confirmed through a confirmatory factor analysis (CFA) with the purpose of verifying whether the two-factor structure fit the sample data. Because of its ordinal nature, the sample size, the number of possible answers (*k* = 5) and the asymmetry and kurtosis values (see Table 1), the CFA was performed using the maximum likelihood estimation method, and the polychoric correlation and asymptotic covariance matrix were used as input.

Model adequacy was analyzed using the chi square over degrees of freedom (χ^2^/df), Non-Normed Fit Index (NNFI), the Comparative Fit Index (CFI), and the Root Mean Square Error of Approximation (RMSEA). NNFI and CFI values over 0.95 indicate a satisfactory fit [41], whereas RMSEA equal to or lower than 0.08 and 0.10 indicate an optimal or satisfactory fit, respectively [42].

The internal consistency of the instrument and its constituent subscales was assessed using Cronbach’s alpha [43], composite reliability (CR), and the average variance extracted (AVE), as well as a correlation analysis between the factors. Convergent validity was analyzed considering that the items loaded strongly on their respective construct, whereas discriminatory validity was analyzed confirming that the AVE of each construct was higher than the squared correlation between the constructs [44].

To determine whether the instrument was invariant across gender groups, a multi-sample CFA was performed. The incremental fit indexes of the alternative models were estimated. The difference between 0.01 or lower between CFI values [45], 0.05 or lower between NNFI values [46], as well as 0.015 or lower RMSEA values indicates insignificant differences [47].

The analyses were carried out using the Statistical Package for the Social Sciences (SPSS) V.23 (IBM, Armonk, NY, USA) and the Linear Structural Relations (LISREL) V.8.80 software [48].

## 4. Results

### 4.1. Descriptive Analysis and Normality

The descriptive data (mean, standard deviation, asymmetry, and kurtosis) for each of the items that compose the sub-scales are shown in Table 1. Asymmetry and kurtosis values were within the range (−1.5 to 1.5), indicating a normal data distribution [49].

### 4.2. Confirmatory Factor Analysis (CFA)

The goodness-of-fit indexes of the two-factor model were satisfactory (χ^2^/df = 2.73, NNFI = 0.94, CFI = 0.95, RMSEA = 0.05). The model showed factorial loads ranging from 0.48 to 0.69 for the autonomy-supportive interpersonal style, whereas the controlling style showed factorial saturations ranging from 0.37 to 0.69 (see Table 1). All the factorial saturations were statistically significant (*p* < 0.05).

### 4.3. Internal Consistency, Correlations, and Convergent and Discriminatory Validity

The reliability analysis revealed that the elimination of neither item improved reliability coefficients, therefore all items from the original version were kept. Results from the reliability analysis yielded alpha values of 0.72 for autonomy support and 0.55 for the controlling style (see Table 2).

The autonomy support subscale presented a composite reliability of 0.77, above the minimum threshold of 0.70 [50]; however, the composite reliability of the controlling style subscale was 0.62, slightly below the aforementioned threshold value. On the other hand, the average variance extracted of the autonomy support subscale was 0.36, and 0.30 for the controlling style, both of which are greater than the squared correlation between both constructs (*r*^2^ = 0.01); therefore, in general, these results support the convergent and discriminatory validity of the instrument (see Table 2).

### 4.4. Factorial Invariance by Gender

To test whether the CEI-EF was invariant across gender groups, a separate CFA was performed for each sample (boys = 254; girls = 246). As shown in Table 3, goodness-of-fit indexes of models for boys (M0a) and girls (M0b) were satisfactory and all estimated parameters were statistically significant (*p* < 0.01).

Later, multi-sample analyses were performed, creating new nested models. Model 1 (M1) examined the structural invariance in the two nested groups showing satisfactory fit indexes, therefore confirming that the factorial structure of the CEI-EF is invariant between the two groups confronted. M1 was used as a baseline for the following nesting of restrictions.

Model 2 (M2) tested the equivalence of the matrix of the factorial saturations across the boys’ and girls’ group. The goodness-of-fit indexes were satisfactory, and the difference obtained between M2 and M1 did not exceed the criterion values; therefore, the invariance in the factorial saturations of the instrument was confirmed in both samples.

Model 3 (M3), which adds the equivalence of the intercepts, showed satisfactory goodness-of-fit indexes. The differences between the goodness-of-fit indexes in the M3 and M1 models did not exceed the criterion values; therefore, the equivalence of the factorial saturations and the intercepts was accepted.

Model 4 (M4) added the invariance of the factorial saturations, intercepts and errors. The M4 results showed satisfactory fit indexes, however, the difference between the CFI and RMSEA values of M4 and M1 exceeded the criterion values; therefore, the strict factorial invariance of CEI-EF across gender could not be confirmed (see Table 3).

## 5. Discussion

The aim of this study was to analyze the psychometric properties, structure, and factorial invariance across gender of the PE teachers’ Interpersonal Styles Questionnaire (CEI-EF) of Sonora, Mexico.

Fit indexes yielded by the CFA were acceptable according to indicators of Barret [51] and were consistent with the results obtained in other studies [18,36]. It is noteworthy that the translation and adaptation of LCQ-S and TCS for Mexico did not require removing any item, contrary to the study by Behzadnia et al. [36], where an item was discarded to attain an appropriate fit of the model; therefore, the Mexican adaptation of the CEI-EF has retained the same number of items as original version of Reeve and Halusic [20].

The internal consistency analysis for the instrument was assessed using Cronbach’s alpha. The LCQ-S yielded an alpha coefficient of 0.72, which is deemed acceptable and consistent with the results obtained from other studies [35]. However, the alpha value obtained for the TCS in this study was 0.55, which did not exceed the criterion value of 0.70 recommended by several authors [52,53]; nonetheless, Schmitt [54] has suggested that there is no general threshold (such as 0.70) for deeming alpha acceptable, but rather that instruments with a significantly low alpha value (0.60 or even 0.50) may be useful in certain circumstances, for example, when a scale is comprised of a small number (e.g., <10) of items [55] or for studies at early research stages [56], as is the case for the instruments used in this study and as is usually the case for empirical psychological studies [57].

The convergent and discriminatory validity of the instrument were assessed using CR and AVE. Subscales of autonomy support and controlling style presented CR values above and very close respectively to the minimum acceptable threshold as established by Hair et al. [50]. On the other hand, in spite of AVE values being below the criterion value, these values were greater than the squared correlation between both constructs [44]; therefore, in general, these results support the convergent and discriminatory validity of the CEI-EF.

One of the largest contributions of this study was to examine the factorial invariance in terms of two groups of different samples and in terms of gender, which had neither been considered in previous studies on the teachers’ Interpersonal Style Questionnaire [20,36], nor on the LCQ [33,39,58], nor on the TCS [34]. Although the strict invariance of the instrument could not be verified, the multi-sample CFA results supported the factorial invariance of factorial saturations and intercepts. According to [59], when a strong factorial invariance is accepted, the mean values of the items and scales are comparable among the groups; therefore, the CEI-EF is an instrument that can measure the perception of autonomy support and controlling style of the PE teacher, and can be used to draw comparisons between boys and girls.

## 6. Limitations

This study has some limitations as well, among which is that all students surveyed came from elementary schools of Sonora, Mexico; therefore, in future research, the sample must be extended to analyze the psychometric properties of the instrument with a population across of different school grades and regions in the country. Another limitation was that no probability sampling design was used during sample selection, thus these results cannot be generalized. Further studies should be carried out proposing different research designs such as, for example, pilot studies with intervention programs for improving both the PE teacher’s skills and the student’s learning process while addressing their basic psychological needs.

## 7. Conclusions

The results support the two-factor structure (controlling style and autonomy support) and the factorial invariance across gender groups of the Mexican version of the CEI-EF, confirming that it is a valid and reliable instrument that can be used by institutions, school centers, school principals, and professors to assess the interpersonal style of teachers and draw comparisons between groups of boys and girls. Teachers can use the CEI-EF to understand the level and interpersonal style perceived by the students during class in order to adjust their teaching practice accordingly. On the other hand, institutions and educational centers may use it as a diagnosis criterion for the selection and recruitment of new teachers who wish to start a career in PE, and as a tool for the continuous evaluation of active teachers with the goal of training and deploying strategies that improve the teacher–student relationship and increase the levels of enjoyment and satisfaction with PE.

## Figures and Tables

**Table 1:** Descriptive and standardized solution of the items and subscales of the instrument.

Sub-Scales		M	SD	A	K	FL
*Autonomy Support* (*Apoyo a la Autonomía*)						
1	…I feel that my teacher provides me with choices and options (…siento que mi profesor me brinda opciones y alternativas)	4.90	2.25	−0.60	−1.05	0.60
2	…I feel understood by my teacher (…me siento comprendido por mi profesor)	5.07	2.03	−0.64	−0.80	0.48
3	… my teacher conveys confidence in my ability to do well in the course (…mi profesor me transmite confianza sobre mi capacidad para realizar bien las actividades durante el curso)	5.23	2.02	−0.81	−0.58	0.69
4	…my teacher encourages me to ask questions (…mi profesor me motiva a que haga las preguntas)	4.42	2.29	−0.21	−1.34	0.49
5	…my teacher listens to how I would like to do things. (…mi profesor escucha como me gustaría hacer las cosas)	4.55	2.22	−0.38	−1.21	0.69
6	…my teacher tries to understand how I see things before suggesting a new way to do things. (…mi profesor trata de comprender como veo las cosas antes de sugerir una nueva forma de hacerlas)	4.71	2.09	−0.44	−1.04	0.65
*Controlling style* (*Estilo controlador*)						
7	…my teacher tries to control everything I do. (...mi profesor trata de controlar todo lo que hago)	4.39	2.34	−0.24	−1.43	0.37
8	… my teacher is inflexible (...mi profesor es inflexible)	3.68	2.36	0.18	−1.47	0.50
9	…my teacher uses forceful language (...mi profesor utiliza un lenguaje fuerte)	3.42	2.37	0.37	−1.39	0.57
10	…my teacher puts a lot of pressure on me. (…mi profesor pone demasiada presión sobre mi)	3.02	2.31	0.65	−1.15	0.69

Note. M = mean; SD = standard deviation; A = asymmetry; K = kurtosis; FL = factorial loadings. All saturations were significant, *t* > 1.96, *p* < 0.05.

**Table 2:** Reliability, bivariate correlations, and discriminant validity between the variables that conform the Interpersonal Styles Questionnaire for Physical Education.

Dimensions	α	CR	AVE	1	2
1. Autonomy support	0.72	0.77	0.36	1	0.01
2. Controlling style	0.55	0.62	0.30	0.07	1

Note. α = Cronbach’s alpha; CR = Composite reliability; AVE = Average variance extracted. The value below the diagonal corresponds to the correlation between the variables. The value above the diagonal corresponds to the squared correlation between the variables.

**Table 3:** Goodness-of-fit indexes of the invariance models.

Model Description		df	SBχ^2^	RMSEA	(90% CI)	NNFI	CFI	ΔNNFI	ΔCFI	ΔRMSEA
M0a	Baseline Model boy	34	67.94 **	0.063	(0.041–0.084)	0.946	0.959			
M0b	Baseline Model girl	34	64.19 **	0.060	(0.037–0.082)	0.924	0.943			
M1	Structural invariance (Baseline Model)	68	92.46 **	0.038	(0.013–0.056)	0.975	0.981			
M2	FL invariance	78	97.25 **	0.031	(0.00–0.050)	0.983	0.985	0.008	0.004	0.007
M3	FL invariance + Int.	86	100.99 **	0.026	(0.00–0.045)	0.988	0.988	0.013	0.007	0.012
M4	FS Invariance + Int. + Error	96	101.27 **	0.014	(0.00–0.037)	0.996	0.996	0.021	0.015	0.024

Note: df = degree of freedom; RMSEA = Root Mean Square Error of Approximation; 90% CI = 90% confidence interval for the RMSEA; NNFI = Non-Normed Fit Index; CFI = Comparative Fit Index; FL = factor load; Int. = intercepts. All comparisons in the Δ indices are made with respect to the baseline model (M1); ** *p* < 0.01.
